# A Novel Approach to Debriefing Medical Simulations: The Six Thinking Hats

**DOI:** 10.7759/cureus.2543

**Published:** 2018-04-27

**Authors:** Xiao Chi Zhang, Hyunjoo Lee, Carlos Rodriguez, Joshua Rudner, Dimitrios Papanagnou

**Affiliations:** 1 Department of Emergency Medicine, Thomas Jefferson University

**Keywords:** faculty development, debriefing, medical simulation, graduate medical education, emergency medicine, innovations in emergency medicine

## Abstract

Simulation has become a standard training method in emergency medicine (EM). Specifically, post-simulation debriefings offer participants the opportunity for reflection while exposing their knowledge and practice gaps. The educational yield of these debriefings, however, is contingent on the debriefer's skills. Without professional development, faculty and educators may not be equipped with supportive debriefing strategies. We propose the Six Thinking Hats (6TH), originally developed by Edward de Bono (1970) as a debriefing framework to support effective, high-yield debriefing conversations. The six colored hats represent six unique approaches to critical thinking. The white hat represents the facts; the green hat, creativity and next steps; the yellow hat, benefits/optimism; the red hat, emotions; the black hat, judgments; and the blue hat, facilitation.

Four junior faculty members underwent a one-hour didactic and one-hour immersive workshop on the 6TH. Two simulation cases were randomly selected from archived simulation cases, which were used for the debriefing process. Each team consisted of one EM resident and one EM faculty. After each simulated case, the facilitator introduced the 6TH at the start of the debriefing, explaining the rules of engagement and the general sequence of hats to be used. Physical hats were worn by the facilitator at the beginning of the session and changed throughout stages of the debriefing, to remind participants of the type of thinking that was taking place at any given time.

Participants who were provided with a colored hat prompt that physically described the type of thinking being employed throughout stages of the debriefing were better able to stay within that respective thinking frame during the discussion, compared to participants who were not provided this visual prompt. Participants of both simulation sessions agreed that the 6TH debriefing style was successful in creating a non-judgmental, comfortable environment that supported open discussion.

The 6TH has the potential to be adopted as a debriefing framework, particularly for junior faculty members without extensive debriefing training. The 6TH is intuitive and has been marked by success in the organizational psychology literature. Faculty development on the 6TH will be essential if this framework is to be used as a debriefing model for educators in health care.

## Introduction

Medical simulation has become a standard pedagogy for training residents across most specialties. Regardless of the level of fidelity used or the task trainers employed, the debriefing conversation following simulations is paramount to resident training and development [1]. As opposed to standard feedback, where an educator provides the learner with targeted information to improve future performance, debriefing calls for a facilitated conversation that must be mindfully choreographed to guide the learners through a safe, reflective dialogue to both identify and close performance gaps [2].

To ensure effective debriefing, facilitators must be able to quickly create a safe, supportive, and respectful learning environment where learners can comfortably share their opinions, thoughts, and experiences [3-7]. The importance of this skill cannot be overstated: as many as 50% of participants may feel intimidated and/or stressed to sit-in on a debriefing for fear of judgement from their educators and peers [8]. Furthermore, simulation educators must also be comfortable at defusing emotionally-charged conversations, redirecting tangential discussions, and drawing parallels to clinical practice without alienating learners [3].

Undeniably, debriefing outcomes significantly rely on the facilitator’s skillset. To assist facilitators with this task, several debriefing methods have been introduced, each with a unique process and set of techniques, to help scaffold the debriefing conversations [3, 9-15]. The sheer variability and complexity of these approaches, however, can be daunting for the novice simulation educator, particularly when one considers the time available for appropriate and effective faculty development.

In light of the aforementioned, the investigators sought to identify an intuitive, easy-to-implement debriefing style for debriefing facilitators, either in the nascent stages of their simulation career, or even seasoned practitioners. We introduce a popular conceptual framework for parallel thinking, the Six Thinking Hats (6TH), developed by Dr. Edward de Bono in 1970, as a feasible debriefing tool for guided post-event debriefing in emergency medicine [16].

## Technical report

The Six Thinking Hats

The Six Thinking Hats, which was originally designed to conduct meetings, support decision-making, and diffuse contentious disputes among team members, utilizes six colored hats to represent six different approaches to critical thinking. The hats are ‘worn’ – one at a time – in consensus. The white hat represents data and information; the green hat represents creativity and new ideas; the yellow hat represents benefits and optimism; the red hat represents feelings and emotions; the black hat represents cautions and judgements; and the blue hat represents the integration, management, and summarization of all of the other hats.

In order for the Six Thinking Hats to work effectively, all members involved in the conversation must agree to comply with the rules of engagement: 1) members must accept ideas from everyone; 2) only one [thinking] hat can be worn at any given time; 3) comments should fit with the thinking framework ascribed with the respective hat color; 4) comments that do not match the hat color should be reserved for the appropriate moment [or hat]; and 5) each comment should lead to a meaningful result.

The purpose of this feasibility study was to determine if the Six Thinking Hats could be used as a debriefing framework – one that is facile enough for educators new to simulation to be able to incorporate into their debriefing practice. The authors posit that this framework has the ability to provide facilitators with the scaffolding needed to conduct and navigate effective, high-yield debriefing conversations. While the Six Thinking Hats has been sporadically referenced in the medical literature with regards to critical thinking [17], to date this is the first report describing the use of de Bono’s Six Thinking Hats as a method to structure post-event debriefing conversations.

Development process

**Figure 1:**
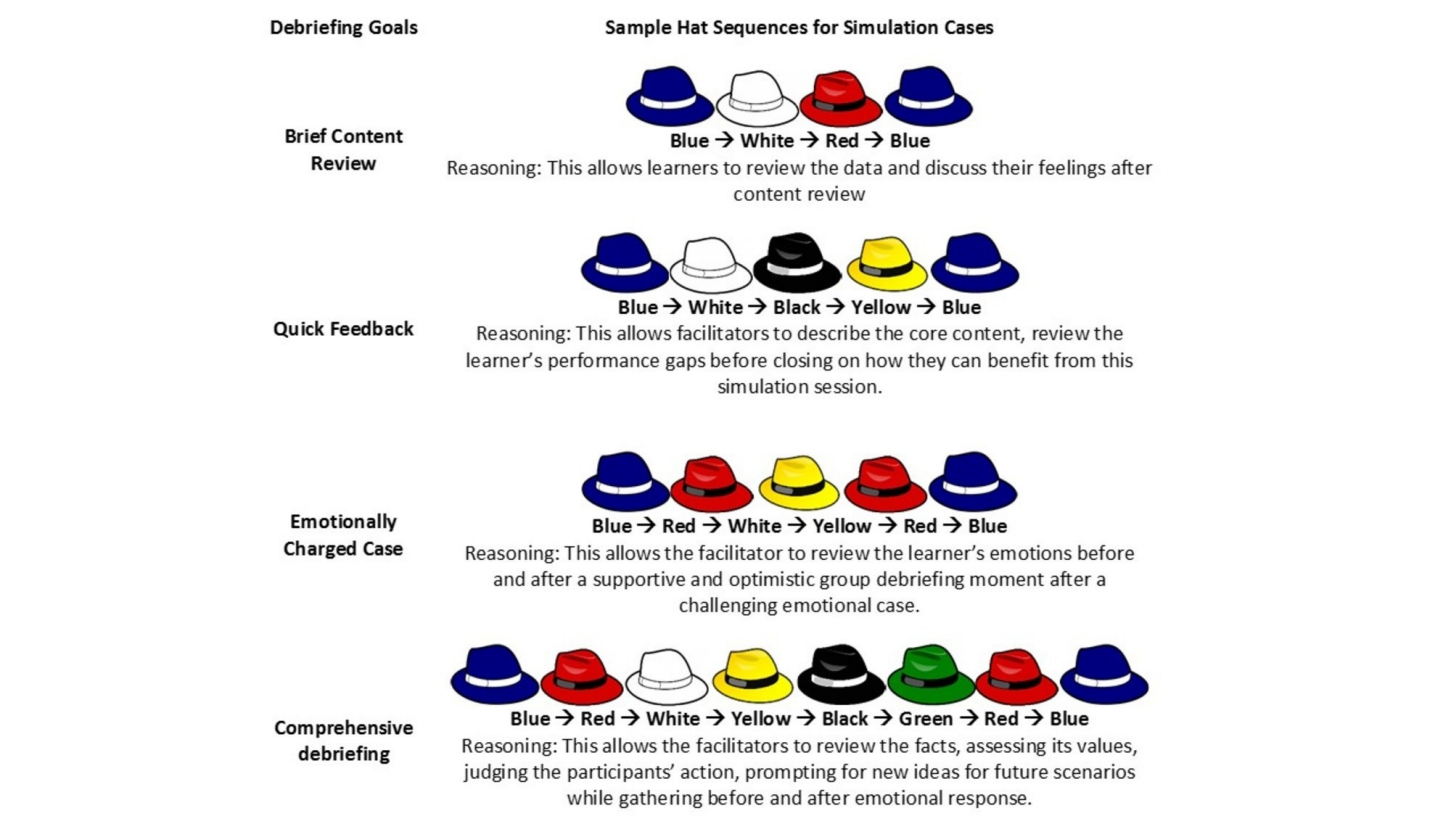
Sample Hat Sequence for Simulation Cases

The authors, who were familiar with the concept of the Six Thinking Hats, ran a series of simulations integrating the hats into resident debriefings. Realizing that simulated cases and clinical encounters will always differ, the authors constructed a list of possible hat sequences with the following debriefing scenarios in mind: a) debriefing for brief content review; b) debriefing for providing concise feedback; c) debriefing the emotionally-charged case; and d) debriefing for a comprehensive review of the case (Figure 1).

The implementation phase

Four junior faculty members (i.e., within one year of completing EM residency) underwent a one-hour didactic and one-hour immersive workshop on the Six Thinking Hats. Faculty training included the basic function of each hat, questions they could ask to promote a dialogue congruent with each respective hat, and sample hat pairings (Table 1).

**Table 1:** De Bono’s 6 Thinking Hats in Simulation Debriefing

Hat Color	Basic Function	Purpose	Questions to Ask	Sample Hat Pairing
White	Data and Information	Be neutral, uncover facts	“What facts do we have or need?”, “How do we get facts?”	Use after [blue] hat to review the facts
Red	Feelings and Emotions	Sharing personal feelings	“How do you feel?” “How are you reacting to this?”	Use after [black] hat to see how people feel
Black	Caution and Judgments	Find weaknesses, make assessments	“Will this work?” “What are its flaws?” “Is this true?”	Use after [white] hat to uncover challenges
Yellow	Benefits and Optimism	Assessing value, extracting benefits, making it work	“What are the benefits?” “How will this help us?” “Why will this work?”	Use after [green] hat to support an idea
Green	Creativity and New Ideas	Make new ideas, new or alternative approach	“What ideas do you have?” “What are other ways to solve this problem?”	Use after [black] or [blue] to generate new ideas
Blue	Integration and Management	Defining goals, make agenda, plan hat-sequence, define outcomes, summarize	“How would you like to approach this?” “Do we know what the case was about?” “How would you summarize this case?”	Start and end with [blue]

Two simulation cases were then randomly selected from the authors' Department of Emergency Medicine residency archives. Case 1 involved managing refractory ventricular tachycardia; Case 2 involved managing a patient with urosepsis. For each case, the facilitator [XCZ] ran the high-fidelity simulation from a control room, separated by a one-way mirror with audio-feedback. Each team consisted of one EM resident and one EM Medical Education fellow [CR and HL] and were tasked to provide appropriate care for the simulated patient. After each simulated case, the facilitator [XCZ] introduced the Six Thinking Hats at the start of the debriefing, introducing the rules of engagement and the sequence of hats that would be used for that particular debriefing. Physical hats (i.e., colored baseball caps) were worn by the facilitator at the beginning of the session and changed throughout stages of the debriefing to remind participants of the type of thinking that was being asked at any given time.

The study investigators who were not directly involved with the simulation/debriefing observed the debriefing encounter in order to effectively evaluate the debriefer [XCZ] upon completion of the case and debriefing. The Quick Feedback Debriefing (Case 1) and the Comprehensive Debriefing (Case 2) sequences were used.

## Discussion

Participants of both sessions universally agreed that this debriefing style created a non-judgmental, comfortable environment that supported open discussion. During the debriefing of Case 1, it was noted that participants had some difficulty staying within the thinking framework for the particular hat being worn; participants occasionally veered off topic and made comments, observations, or statements that would have been better suited for a different hat. The Debriefing of Case 1 revealed that even though the colored hat was physically worn by the debriefer, participants would forget its affiliated function.

To address this, for the debriefing of Case #2, the crown of each hat displayed a keyword that represented the thinking style associated with that respective hat. During this iteration, participants were better able to stay within the respective thinking hats. At one point, when one of the resident participants went off topic, the other participants gently reminded the participant that his comment was not congruent with the thinking hat being worn, prompting him to share this comment later in the debriefing.

The Six Thinking Hats offers a feasible and facile approach to debrief simulations, particularly for junior faculty and individuals new to debriefing and simulation. Residents (i.e., participants) and fellows (i.e., debriefers) appreciated the opportunity to participate in both cases and undergo this type of debriefing. The debriefing was facile enough for residents to follow. Similarly, the facilitators appreciated having the ability to use prompts to transition the conversation.

While this is only a report of feasibility, future studies should identify ideal hat sequences for simulated debriefings. Additionally, debriefings employing the Six Thinking Hats should be evaluated and rated by expert debriefers using several validated rater forms [18]. The authors recommend future iterations of debriefings using the Six Thinking Hats with visual prompts (i.e., posters, handouts) depicting the Six Thinking Hats, as well as the proposed hat order for the debriefing (Figure 1); this will help participants better understand the flow of the debriefing conversation.

## Conclusions

The Six Thinking Hats approach builds on adult learning theory, simulation education, and critical thinking, and creates an easy-to-apply method for simulation educators as they tackle the complex art of debriefing. The proposed framework has the potential to empower both simulation educators and participants to have valuable debriefing conversations that can promote learning and reflection. Faculty development on the Six Thinking Hats will be essential if this framework is to be used as a debriefing model for educators in health care.
